# Dose-Dependent Effects of Long-Term Administration of Hydrogen Sulfide on Myocardial Ischemia–Reperfusion Injury in Male Wistar Rats: Modulation of RKIP, NF-κB, and Oxidative Stress

**DOI:** 10.3390/ijms21041415

**Published:** 2020-02-19

**Authors:** Sajad Jeddi, Sevda Gheibi, Khosrow Kashfi, Mattias Carlström, Asghar Ghasemi

**Affiliations:** 1 Endocrine Physiology Research Center, Research Institute for Endocrine Sciences, Shahid Beheshti University of Medical Sciences, Tehran 19395-4763, Iran; Sajad.jeddi@sbmu.ac.ir (S.J.); sevda.1365@yahoo.com (S.G.); 2 Department of Molecular, Cellular and Biomedical Sciences, Sophie Davis School of Biomedical Education, City University of New York School of Medicine, New York, NY 10031, USA; 3 Department of Physiology and Pharmacology, Karolinska Institute, 17177 Stockholm, Sweden; mattias.carlstrom@ki.se

**Keywords:** hydrogen sulfide, nitric oxide, infarct size, ischemia–reperfusion injury, H_2_S-producing enzymes, NO-producing enzymes, RKIP, NF-κB, oxidative stress

## Abstract

Decreased circulating levels of hydrogen sulfide (H_2_S) are associated with higher mortality following myocardial ischemia. This study aimed at determining the long-term dose-dependent effects of sodium hydrosulfide (NaSH) administration on myocardial ischemia-reperfusion (IR) injury. Male rats were divided into control and NaSH groups that were treated for 9 weeks with daily intraperitoneal injections of normal saline or NaSH (0.28, 0.56, 1.6, 2.8, and 5.6 mg/kg), respectively. At the end of the study, hearts from all rats were isolated and hemodynamic parameters were recorded during baseline and following IR. In isolated hearts, infarct size, oxidative stress indices as well as mRNA expression of H_2_S-, nitric oxide (NO)-producing enzymes, and inflammatory markers were measured. In heart tissue following IR, low doses of NaSH (0.28 and 0.56 mg/kg) had no effect, whereas an intermediate dose (1.6 mg/kg), improved recovery of hemodynamic parameters, decreased infarct size, and decreased oxidative stress. It also increased expression of cystathionine γ-lyase (CSE), Raf kinase inhibitor protein (RKIP), endothelial NO synthase (eNOS), and neuronal NOS (nNOS), as well as decreased expression of inducible NOS (iNOS) and nuclear factor kappa-B (NF-κB). At the high dose of 5.6 mg/kg, NaSH administration was associated with worse recovery of hemodynamic parameters and increased infarct size as well as increased oxidative stress. This dose also decreased expression of CSE, RKIP, and eNOS and increased expression of iNOS and NF-κB. In conclusion, chronic treatment with NaSH has a U-shaped concentration effect on IR injury in heart tissue. An intermediate dose was associated with higher CSE-derived H_2_S, lower iNOS-derived NO, lower oxidative stress, and inflammation in heart tissue following IR.

## 1. Introduction

Myocardial ischemia (MI) is one of the leading causes of cardiovascular morbidity and mortality worldwide [1]. MI often occurs following a partial or complete occlusion of the coronary arteries, and while reperfusion rescues the ischemic heart from expected death, it is associated with ischemia-reperfusion (IR) injury [2]. Protective effects of ischemic pre-and post-conditioning against myocardial IR injury have been documented in animals [3]; clinical translation of these results has however not been very successful [4,5,6]. Another approach for protecting the heart against ischemia is using pharmacological agents, which appears to be a more realistic and feasible approach from the clinical perspective [7].

Hydrogen sulfide (H_2_S) is produced in the cardiomyocytes by at least three H_2_S-producing enzymes, i.e., cystathionine β-synthase (CBS), cystathionine γ-lyase (CSE), and 3-mercaptopyruvate sulfurtransferase (3-MST), of which CSE is the most important one [8]. In mice, CSE deficiency decreases tolerance to IR injury [9] and overexpression of the enzyme attenuates myocardial IR injury [10]. Protective effects of H_2_S against myocardial IR injury are nitric oxide (NO)-dependent [9,11,12]. In endothelial NO synthase (eNOS)-knockout mice, administration of H_2_S donors failed to protect the heart from IR injury [9]. eNOS-derived NO protected against myocardial IR injury [13,14]; while inducible NOS (iNOS)-derived NO contributed to IR injury [15,16]. Inhibition of CSE in mice exacerbated myocardial IR injury by decreasing eNOS-derived NO [9] and increasing iNOS-derived NO [17]. Administration of H_2_S donors increased tolerance to myocardial IR in rodents by decreasing iNOS expression [18,19] and increasing eNOS activation [9].

IR injury starts with oxidative stress and inflammation, followed by apoptosis and necrosis leading to irreversible cell death [20]. The Raf kinase inhibitory protein (RKIP) expression is associated with inflammation-induced diseases [21]. Loss of RKIP increases the nuclear factor kappa-B (NF-κB) transcription factor, whereas overexpression of RKIP reduces it [22]. Activation of NF-κB promotes inflammation in the setting of myocardial ischemia and exacerbates the heart’s response to IR injury [23]. Anti-inflammatory effects of sodium hydrosulfide (NaSH) have been reported after short-term administration before myocardial IR [24,25]; however, the effects of NaSH on RKIP and NF-κB have not been reported.

In fact, results of a recent meta-analysis in normal rats and mice have shown that exogenous H_2_S administration has a protective effect against myocardial IR injury [26]. The cardioprotective effects of H_2_S-releasing agents have been studied in vitro [26,27,28,29,30,31,32]. So far, the in vivo studies are mostly short-term where NaSH has been administrated 15 min [31], 6 days [32], or 7 days [28,29,30] before ischemia. To our knowledge, there is no long-term study assessing the dose-dependent effects of H_2_S on the cardiovascular system. In addition, a biphasic response to exposure to increasing H_2_S has been reported in brain; i.e., NaSH in the low-to-intermediate doses could protect the brain from IR injury, while at higher doses opposite effects were observed [33]. Therefore, the aim of this study was to determine dose-dependent, long-term in vivo effects of NaSH administration on myocardial IR injury in male rats and to evaluate and correlate RKIP, NF-κB, and oxidative stress responses under these conditions.

## 2. Results

### 2.1. Effect of NaSH Administration on Body and Heart Weights

The body weights of animals were similar in all assigned groups before starting the experiments (Table 1). NaSH administration for 9 weeks at 0.28–1.6 mg/kg had no significant effects on body weights; however, at 5.6 mg/kg, it significantly increased heart weight (*p* = 0.0002), and heart-to-body-weight ratio (*p* = 0.0004) as assessed at the end of study.

### 2.2. Effect of NaSH on Systolic Blood Pressure, Heart Rate, and Hemodynamic Parameters

**Figure 1:**
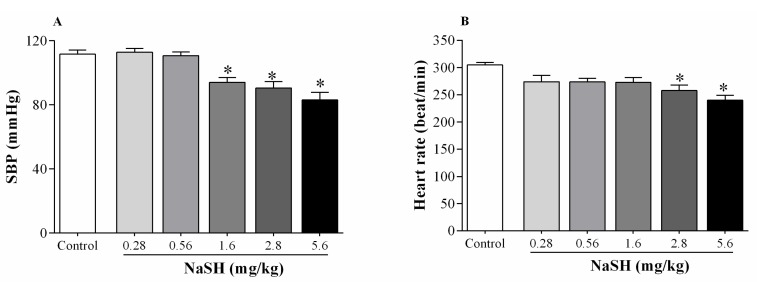
Dose-dependent effects of NaSH on systolic blood pressure (SBP) (**A**) and heart rate (**B**). Values are mean ± SEM (n = 6/group); * *p* < 0.05 compared to non-treated control rats.

NaSH administration at 1.6, 2.8, and 5.6 mg/kg decreased systolic blood pressure (SBP) (Figure 1A); heart rate was decreased at 2.8 and 5.6 mg/kg (Figure 1B).

**Figure 2:**
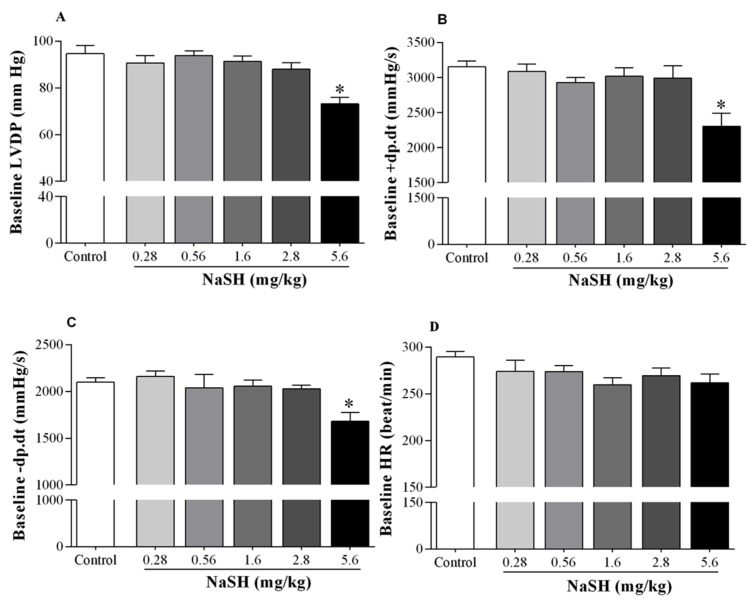
Effects of different doses of NaSH on hemodynamic parameters during the stabilization period. Hemodynamic parameters included left ventricular developed pressure (LVDP, **A**); peak rate of positive changes in left ventricular pressure (+dp/dt, **B**); peak rate of negative changes in left ventricular pressure (−dp/dt, **C**); and heart rate (HR, **D**). Values are mean ± SEM (n = 6/group); * *p* < 0.05 compared to non-treated control rats.

During the stabilization period, NaSH at 5.6 mg/kg decreased left ventricular developed pressure (LVDP) from 94.7 ± 3.5 to 73.2 ± 2.7 mmHg (*p* = 0.0001) (Figure 2A), peak rate of positive changes in left ventricular pressure (+dp/dt) from 3153 ± 84 to 2307 ± 185 (*p* = 0.0012) (Figure 2B), and peak rate of negative changes in left ventricular pressure (−dp/dt) from 2099 ± 50 to 1682 ± 93 (*p* = 0.0180) (Figure 2C). At all doses tested, NaSH had no effects on baseline heart rates (Figure 2D).

**Figure 3:**
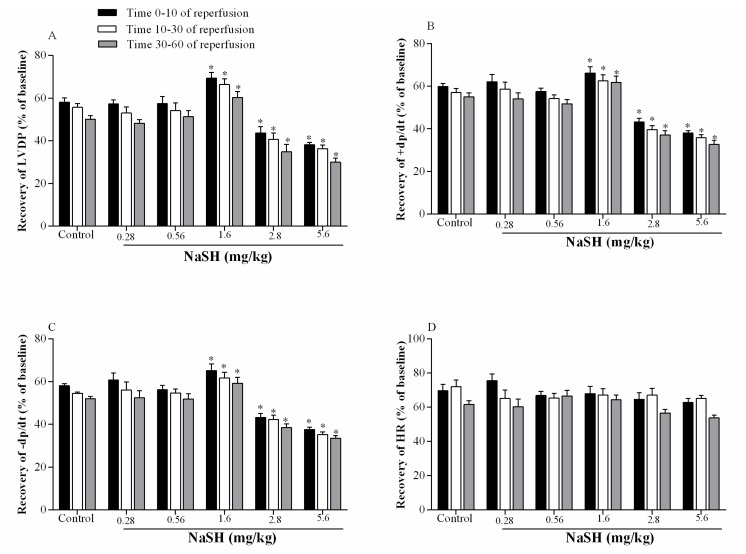
Effects of different doses of NaSH on hemodynamic parameters during the recovery period. Hemodynamic parameters included left ventricular developed pressure (LVDP, **A**); peak rate of positive changes in left ventricular pressure (+dp/dt, **B**); peak rate of negative changes in left ventricular pressure (−dp/dt, **C**) and heart rate (HR, **D**). Values are mean ± SEM (n = 6/group); * *p* < 0.05 compared to non-treated control rats.

Compared to controls, NaSH at 0.28 and 0.56 mg/kg had no effect on recoveries of LVDP (Figure 3A), +dp/dt (Figure 3B), and −dp/dt (Figure 3C) following ischemia; however, it significantly increased recoveries of LVDP, +dp/dt, and −dp/dt at 1.6 mg/kg and decreased these parameters at 2.8 and 5.6 mg/kg, Figure 3A–C. Heart rate recovery was not affected at any dose, Figure 3D.

### 2.3. Effect of NaSH on H_2_S and Nitrite + Nitrate (NOx) Levels in Heart Tissue

**Figure 4:**
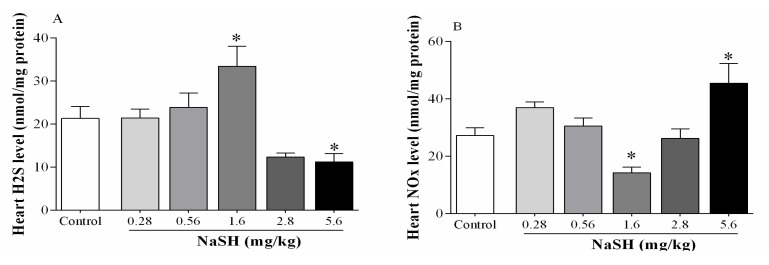
Effects of different doses of NaSH on heart hydrogen sulfide (H_2_S, **A**) and nitrite + nitrate (NOx, **B**) levels after the ischemia–reperfusion (IR) period. Values are mean ± SEM; (n = 6/group); * *p* < 0.05 compared to non-treated rats.

Following IR, H_2_S levels in the heart increased from 21.3 ± 2.7 to 33.4 ± 4.6 nmol/mg protein (*p* = 0.0274) when NaSH was administered at 1.6 mg/kg; at lower doses and at 2.8 mg/kg, there were no effects on H_2_S levels; however, at 5.6 mg/kg, H_2_S levels decreased to 11.2 ± 1.9 nmol/mg protein (*p* = 0.0920) Figure 4A.

**Figure 5:**
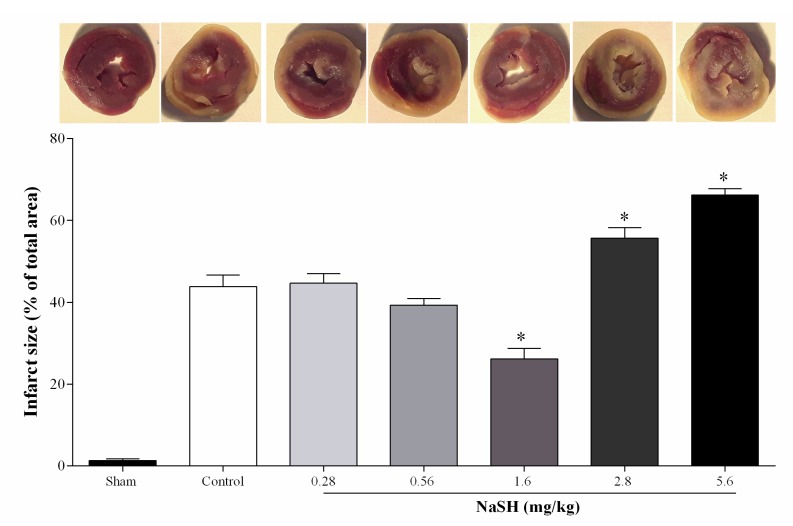
Effects of different doses of NaSH on infarct sizes. Values are mean ± SEM; (n = 6/group); * *p* < 0.05 compared to non-treated control rats.

NOx levels decreased from 27.2 ± 2.7 to 14.2 ± 1.9 nmol/mg protein (*p* = 0.0906) at 1.6 mg/kg NaSH (Figure 5B), but NOx levels increased to 45.4 ± 6.9 nmol/mg protein (*p* = 0.0077) at 5.6 mg/kg NaSH, with no effect at any of the other doses, Figure 4B.

### 2.4. Effect of NaSH on Infarct Size

NaSH at 0.28 and 0.56 mg/kg had no effect on myocardial infarct size, while at 1.6 mg/kg this was decreased by 40% (*p* < 0.0001), and at 2.8 and 5.6 mg/kg this was increased by 27% (*p* = 0.0024) and 51%, (*p* < 0.0001), respectively (Figure 5).

### 2.5. Effect of NaSH on mRNA Expression of H_2_S- and NO-Producing Enzymes in the Heart

**Figure 6:**
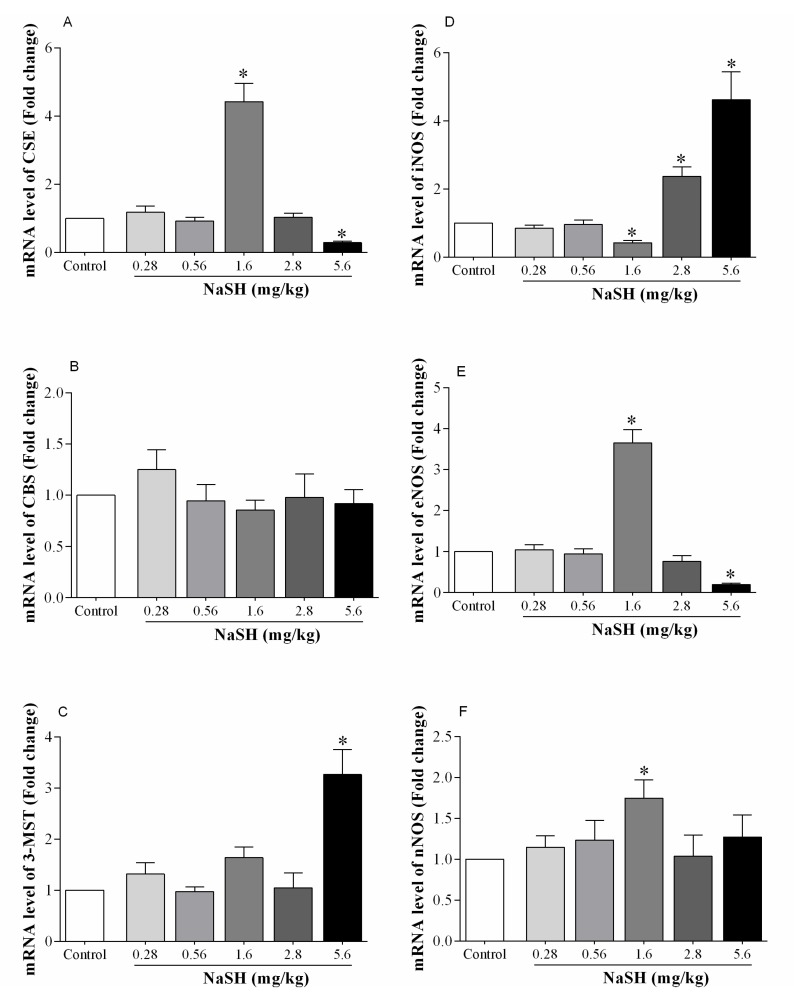
Effect of NaSH on mRNA expression of H_2_S- and NO-producing enzymes in heart tissue. H_2_S-producing enzymes including cystathionine gamma-lyase (CSE, **A**), cystathionine-β-synthase (CBS, **B**), and mercaptopyruvate sulfurtransferase (3-MST, **C**) and NO-producing enzymes including inducible nitric oxide synthase (iNOS, **D**), endothelial nitric oxide synthase (eNOS, **E**), and neuronal nitric oxide synthase (nNOS, **F**). Values are mean ± SEM; (n = 6/group); * *p* < 0.05 compared to non-treated control rats.

Following ischemia, mRNA expression for CSE increased by 342% at a NaSH dose of 1.6 mg/kg, while this was decreased by 71% at 5.6 mg/kg; at other doses, CSE expression was not significantly different from that of the controls (Figure 6A).

NaSH had no effect on mRNA expression of CBS at any of the doses employed (Figure 6B). The level of 3-MST mRNA was increased by 220% at 5.6 mg/kg NaSH with no effects at any of the other doses (Figure 6C).

Following IR, NaSH at 1.6 mg/kg decreased mRNA expression of iNOS by 58% (*p* < 0.0001), that of eNOS and neuronal NOS (nNOS) were increased by 265% (*p* < 0.0001) and 75% (*p* = 0.0225), respectively, Figure 6D–F. NaSH at 2.8 mg/kg increased iNOS expression by 204% (*p* < 0.0001), and at 5.6 mg/kg by 362% (*p* < 0.0001). NaSH at 2.8 mg/kg did not have an effect on eNOS expression; however, at 5.6 mg/kg it decreased it by 81% (*p* < 0.0001). NaSH at 0.28 and 0.56 mg/kg had no effect on mRNA expression of the NO-producing enzymes.

### 2.6. Effect of NaSH on mRNA Expression of Inflammation-Related Markers in the Heart

**Figure 7:**
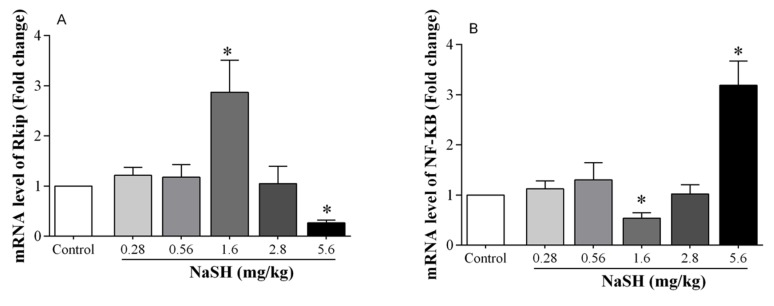
Effect of NaSH on mRNA expression of Raf kinase inhibitor protein (RKIP, **A**) and nuclear factor kappa-B (NF-κB, **B**) in heart tissue. Values are mean ± SEM; (n = 6/group); * *p* < 0.05 compared to non-treated control rats.

RKIP expression was increased by 187% (*p* = 0.0260) at a NaSH dose of 1.6 mg/kg, and it was decreased by 73% (*p* < 0.0001) at a dose of 5.6 mg/kg, Figure 7A. NaSH at a dose of 1.6 mg/kg decreased mRNA expression of NF-κB by 46% (*p* = 0.0018), whereas at 5.6 mg/kg it increased it by 219% (*p* < 0.0001), Figure 7B. Other doses of NaSH had no effect on RKIP or NF-κB expression.

### 2.7. Effect of NaSH on Oxidative Stress Indices in Heart Tissue

**Figure 8:**
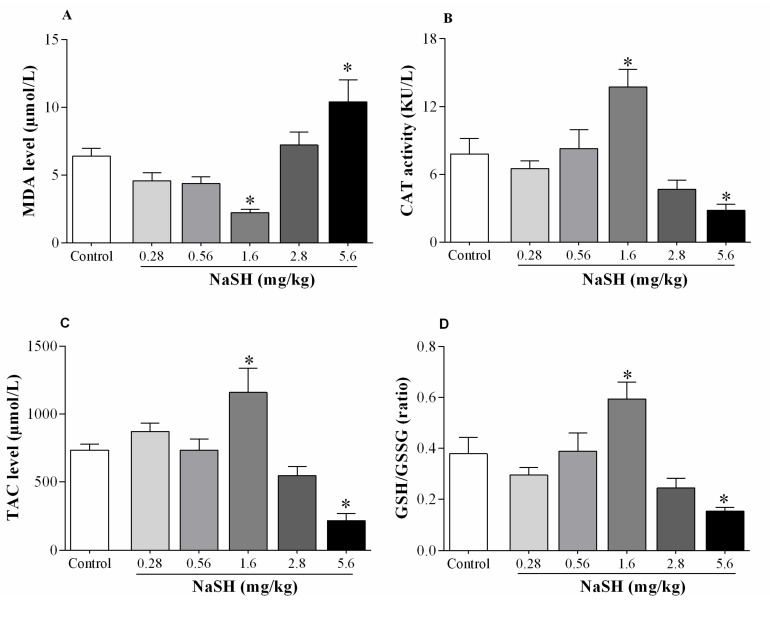
Effects of NaSH administration on oxidative stress indices of heart tissue. Oxidative stress indices including malondialdehyde (MDA, **A**), catalase activity (CAT, **B**), total antioxidant capacity (TAC, **C**), and reduced glutathione-to-oxidized-glutathione ratio (GSH/GSSG, **D**). Values are mean ± SEM; (n = 6/group); * *p* < 0.05 compared to non-treated control rats.

Following IR, compared to the non-treated rats, NaSH at 1.6 mg/kg decreased malondialdehyde (MDA) levels by 65% (*p* = 0.0303), Figure 8A; increased catalase (CAT) activity by 76% (*p* = 0.0228), Figure 8B; increased total antioxidant capacity (TAC) concentration by 58% (*p* = 0.0408), Figure 8C; and increased reduced glutathione (GSH)/oxidized glutathione (GSSG) ratio by 57% (*p* = 0.0948), Figure 8D.

NaSH at 5.6 mg/kg increased MDA levels by 62% (*p* = 0.0437), Figure 8A; decreased CAT activity by 64% (*p* = 0.0938), Figure 8B; decreased TAC concentration by 71% (*p* = 0.0064), Figure 8C; and decreased GSH/GSSG ratio by 59% (*p* = 0.0690), Figure 8D. Other doses of NaSH had no effects on any of these parameters.

## 3. Discussion

Our results showed a biphasic effect of NaSH on myocardial IR injury in normal rats. At an intermediate dose (1.6 mg/kg), NaSH had a protective effect against IR; at low doses (0.28 and 0.56 mg/kg), it had no effect; and at a high dose (5.6 mg/kg), it exacerbated myocardial IR injury. Favorable effects of NaSH at the intermediate dose of 1.6 mg/kg on cardiac function were, at least in part, associated with increased CSE expression, which is in line with the higher measured cardiac H_2_S levels, higher eNOS expression, and lower iNOS expression, which is also in line with the lower levels of cardiac NO and attenuated IR-induced oxidative stress and inflammation. NaSH at the highest dose tested, 5.6 mg/kg, had the opposite effects.

In this study, NaSH at doses of ≥1.6 mg/kg decreased SBP, and, at a dose ≥2.8 mg/kg, it decreased heart rate in the whole intact animal. What should be emphasized is that under physiological conditions, a decrease in blood pressure is accompanied with an increase in heart rate, this phenomenon is dampened with NaSH administration. Thus, in the long run, H_2_S may lead to cardiac remodeling, which may prove to be detrimental to the overall cardiac function. At what dose or dose-range, this remodeling may occur is not currently apparent and needs further long-term studies. In line with our results, a reduction in SBP and heart rate was observed in rats when NaSH was administered at a dose of 5.6 mg/kg for 28 days [34]. The underlying mechanism(s) for the reduction in SBP and heart rate in response to H_2_S-releasing agents is not clear; however, these are most likely not mediated through the activation of the parasympathetic nervous system, ATP-sensitive K^+^ and/or L-type voltage-sensitive Ca^2+^ channels [35,36]. Reductions in heart rate could be due to the inhibitory effects of H_2_S on the metabolism [37]; we recently reported that long-term administration of NaSH to rats perturbed carbohydrate metabolism [38].

NaSH at a dose of 5.6 mg/kg caused an increase in the weight of the hearts. This increase may be associated with lower cardiac contractility and is congruent with decreased baseline LVDP and ±dp/dt that was observed in our study. In addition, increased heart weight can be attributed to the vasodilatory effects of NaSH, since vasodilators at high doses can increase heart weight in normal mice [39].

In this study, NaSH at a dose of 1.6 mg/kg decreased infarct size and increased recoveries of LVDP and ±dp/dt, thus exhibiting a protective effect. Dose-dependent effects of short-term NaSH administration against myocardial IR injury in normal rats have been assessed both in vivo [40] and in vitro [41]; results indicate that NaSH at an intermediate dose (1.6 mg/kg) has protective effects whereas lower doses and higher doses have no effects on myocardial IR injury. Similarly, Kang et al. [28] reported that NaSH at 1.6 mg/kg increases tolerance against myocardial IR injury in normal rats. Our results regarding the protective effects of NaSH administration for 63 days extend previous short-term studies of NaSH administration that were for 5 days [42], 6 days [32], and 7 days [29,30] before inducing ischemia. In these studies, NaSH was administered at a dose of 0.78 mg/kg, which showed decreases in infarct size following myocardial IR injury.

Regarding the relatively higher doses of NaSH (2.8 and 5.6 mg/kg), in our study, NaSH decreased recoveries of hemodynamic parameters following myocardial IR injury and increased infract size; This is in contrast to some reported studies where NaSH at 3 mg/kg, 15 min before ischemia and at 5.6 and 16.8 mg/kg, 10 min before ischemia, had a protective effect [24,31] or no effect [40] against myocardial IR injury in normal rats. This apparent discrepancy may be due to the duration of NaSH administration, which in our study was 63 days, as toxic effects of H_2_S are dependent on both dose and duration of exposure [43].

As summarized in Table 2 [18,24,28,29,30,31,32,40,42,44,45,46], we did not find any studies that addressed the long-term in vivo effects of an H_2_S-releasing agent(s) on cardiac function; all studies reported were for up to 1 week of treatment. In addition, there are only a handful of studies that have addressed the dose-dependent in vivo effects of NaSH on cardiac tolerance against IR injury. Cardioprotective effects of NaSH have mostly been reported for short-term (up to 7 days) daily injections of NaSH, or for a single dose shortly before ischemia. In general, H_2_S-releasing agents have shown biphasic effects on IR injury using perfused hearts [10,40,47] with a very narrow therapeutic window [26,48]. Favorable effects of H_2_S are observed at low-to-intermediate concentrations, while detrimental effects are observed at high concentrations [43]. Our data are in line with studies that have suggested protective effects at low-to-intermediate concentrations of H_2_S (<10 μM) in the heart, while higher concentrations (>10 μM) have deleterious effects [43]. Thus, determining the appropriate dose of H_2_S is a critical issue for the development of H_2_S-based therapeutics [48].

In this study, we showed that NaSH at 1.6 mg/kg increased CSE expression, while at 5.6 mg/kg it decreased the same, these correlated well with the measured H_2_S levels. CSE is the most important H_2_S-producing enzyme in the cardiovascular system [49], and cardiac H_2_S levels decrease ~80% in CSE knockout mice [50]. Here, administration of NaSH at 5.6 mg/kg lowered mRNA expression of cardiac CSE by ~70%, which may explain in part the lower cardiac H_2_S levels of ~50% that were observed in our study. We are not aware of any studies addressing the chronic in vivo effects of NaSH administration on H_2_S-producing enzymes in the heart after IR; however, lower CSE expression in cardiomyocytes has been reported in vitro with several H_2_S-releasing agents at high doses [51]. Most often data obtained from in vitro studies cannot be directly applied to predict the response of a whole organism [52,53] since in vitro studies do not repeat or represent the whole animal physiology [54]. In our study, tolerance to IR injury correlated well with CSE expression in the heart tissue. In support of these data, CSE knockout [9] or pharmacologic inhibition of CSE [55,56] decreased tolerance to IR injury in rats. In addition, CSE overexpression in mice increases tolerance to myocardial IR injury [10]. In our study, NaSH at 5.6 mg/kg increased 3-MST expression, we propose that this may be in compensation for the observed decreased CSE expression as has been previously reported for heart tissue after IR in mice [57].

In this study, NaSH at 1.6 mg/kg increased eNOS and nNOS expressions and it decreased iNOS expression following myocardial IR; while at 5.6 mg/kg it increased iNOS and decreased eNOS expressions with effectively no changes in nNOS. Thus, the increased cardiac NOx levels observed in our study after IR, could be ascribed to an increase in iNOS activity. In our study, enzyme activity or expression was not measured, but an elevated mRNA expression for iNOS was found. Higher NOx levels in the heart tissue are an important factor for increasing IR injury [58] as it increases lipid peroxidation [59] and nitrosative stress [60]. In our study, decreased and increased iNOS/eNOS ratios were associated with higher and lower tolerance against myocardial IR, respectively. In this regard, it has been reported that eNOS- and nNOS-derived NO [13,16,61] has protective roles against myocardial IR injury; however iNOS-derived NO contributes to myocardial IR injury [15,62] and is also accompanied by cardiac hypertrophy [63] and oxidative stress [64]. Both detrimental effects were observed in our study following NaSH administration at the high dose. In line with our results, it has been reported that low-to-intermediate doses of NaSH [18] and diallyl trisulfide present in garlic [19], prior to reperfusion provide tolerance against myocardial IR by decreasing iNOS expression in rats and mice. It has also been reported that CSE-derived H_2_S modulates NOS activity [65,66], e.g., inhibition of CSE in mice decreases eNOS-derived NO [9] and increases iNOS-derived NO [17].

In this study, NaSH at 1.6 mg/kg decreased markers of oxidative stress in the heart tissue after IR, while at 5.6 mg/kg it increased them. In line with our results, decreased MDA levels in heart tissues have been reported following short-term in vivo NaSH administration at low dose (0.78 mg/kg/day for 5 days before IR) [42]. Low dose of NaSH has been suggested to inhibit oxidative stress by increasing SOD activity [67], decreasing ROS levels [68], increasing expression or activity of eNOS [69] and CSE [70], while at high doses it increases oxidative stress by increasing ROS as well as decreasing GSH levels [71,72].

Finally, we showed that NaSH at 1.6 mg/kg increased mRNA expression of RKIP and decreased the mRNA expression of NF-κB in the heart tissue following IR, while at 5.6 mg/kg, it had the opposite effects. The effect of NaSH on mRNA level of RKIP in a setting of IR injury has not been previously reported; however the positive effect of NaSH on protein kinase C (PKC) activation [73] (upstream pathway of RKIP) and negative effects on mitogen-activated protein kinase (MAPK) activation and NF-κB translocation [24] (downstream pathways of RKIP) following myocardial ischemia have been reported. PKC and MAPK have protective and detrimental effects against myocardial IR injury, respectively [24,73]. Anti-inflammatory effects of NaSH have been reported following short-term administration (i.e., 15 min and 7 days) at doses of 3 mg/kg [24] and 0.78 mg/kg [25] before myocardial IR. In addition, the biphasic effects of H_2_S on inflammatory signaling have also been observed in LPS-treated murine macrophages; NaSH at low doses decreases NF-κB activity, but at high doses, it increases the synthesis of proinflammatory mediators and NF-κB activity [74]. These biphasic or U-shaped effects of chronic NaSH administration on oxidative stress and inflammation indices provide further evidence of a protective effect of intermediate dose and detrimental effect of high dose of H_2_S-releasing agent(s).

As a strength, we evaluated multiple long-term doses of NaSH administered in an in vivo setting on tolerance against myocardial IR injury. The dose–response design has been reported to be one of the most important criteria that would increase the chance of an animal study to be translated from the bench to the bedside [75]. In addition, given that a living day in a rat is equivalent to 26 days in a human [76], 9 weeks of NaSH administration in our rat studies could be considered as a long-term intervention in a human. However, further studies are needed to determine time-dependent effects of H_2_S donors on myocardial IR injury.

As a limitation, H_2_S concentrations were measured by the methylene blue method, which measures all sulfur species rather than only free H_2_S. We did not evaluate the effects of our NaSH intervention on the various parameters by Western blots, due to lack of resources. However, at least for our measurements of H_2_S and NO levels, our mRNA data correlate well with these measured values, and we may infer that these would also be in line with protein expressions as well.

## 4. Materials and Methods

### 4.1. Animals

Male Wistar rats (190–210 g) were housed under controlled conditions (23 ± 2 °C, 12/12 h light–dark cycle, relative humidity of 50% ± 6%) with food and water ad libitum. All experimental procedures employed, as well as caring and handling of the rats, were approved and performed in accordance with guidelines provided by the local ethics committee of the Research Institute for Endocrine Sciences of Shahid Beheshti University of Medical Sciences (IR.SBMU.ENDOCRINE.REC.1398.036, 6 August, 2019).

### 4.2. Experimental Design

**Figure 9:**
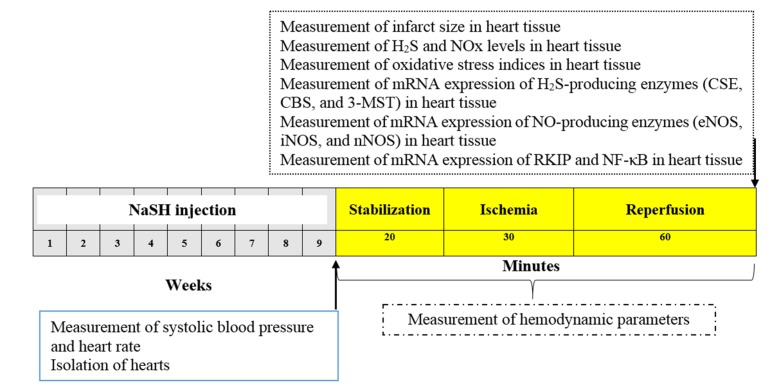
Experimental protocol and the timeline of the study. NaSH at 0.28, 0.56, 1.6, 2.8, and 5.6 mg/kg/day was administrated for a period of 9 weeks as detailed in Section 2.2. NaSH, sodium hydrosulfide; H_2_S, hydrogen sulfide; CBS, cystathionine β-synthase; CSE, cystathionine γ-lyase; 3-MST, 3-mercaptopyruvate sulfurtransferase; NOS, nitric oxide synthase; nNOS, neuronal NOS; eNOS, endothelial NOS; iNOS, inducible NOS; RKIP, Raf kinase inhibitor protein; NF-κB, nuclear factor kappa-B.

The experimental protocol is shown in Figure 9. Male rats were divided into control group (n = 6) and NaSH (0.28, 0.56, 1.6, 2.8, and 5.6 mg/kg/day) groups (n = 6/group). The control group received intraperitoneal (IP) injections of normal saline and the NaSH groups received IP injection of 0.28, 0.56, 1.6, 2.8, and 5.6 mg/kg/day of NaSH, freshly prepared each day, for 9 weeks. Body weights (using Tefal Scale; sensitivity 1 g) were recorded at the start and end of the interventions. At the end of study, systolic blood pressure and heart rate were measured in rats using a noninvasive tail-cuff method (AD Instruments, MLT125R, New South Wales, Australia). Systolic blood pressure and heart rate values were averaged from three consecutive recordings obtained from each rat. At week 9, hearts from all rats were isolated and connected to a Langendorff apparatus and hemodynamic parameters’ (LVDP, +dP/dt and −dp/dt) change in left ventricular pressure were recorded both at baseline (stabilization period) and also during IR. In addition, the weights of the hearts, levels of H_2_S, NOx, MDA, TAC, GSH, total glutathione (GSH + GSSG), CAT activity, and infarct size were measured in all groups after the IR period. We also measured the mRNA expression levels of CSE, CBS, 3-MST, eNOS, iNOS, nNOS, RKIP, and NF-κB in the heart tissue following IR.

### 4.3. Measurement of Hemodynamic Parameters

Details for the measurements of the heart rate, LVDP, and ±dp/dt by the Langendorff apparatus were previously described [77]. In brief, at the end of the interventions, all rats were anesthetized with an IP injection of ketamine/xylazine (50/10 mg/kg) and the hearts were quickly removed. Isolated hearts were immersed in ice-cold perfusion buffer, the aortae were rapidly cannulated and connected to the Langendorff apparatus. A retrograde perfusion was performed with Krebs–Henseleit solution (KHS). Composition of KHS in mM was: 118.6 NaCl; 4.7 KCl; 2.5 CaCl_2_; 1.6 MgSO_4_; 1.2 KH_2_PO_4_; 25 NaHCO_3_; 11.1 glucose (all from Merck, Darmstadt, Germany), equilibrated with 95% O_2_:5% CO_2_, (pH 7.4). For measurement of the heart rates, LVDP, ±dp/dt, and LVEDP, a latex balloon was inserted into the left ventricle and LVEDP was adjusted at 5–10 mmHg in all hearts by filling the latex balloon with water. Isolated hearts were subjected to 20 min of stabilization, 30 min of global ischemia, and 60 min of reperfusion, respectively. LVEDP, LVDP, and ±dp/dt were digitalized by a data acquisition system (Power Lab, AD instrument, Australia). At the end of the reperfusion phase, the isolated hearts were separated from the Langendorff apparatus, weighed, and stored at −80 °C for later analyses.

### 4.4. Measurements of H_2_S and NOx Levels in Heart Tissues

At the end of study, tissue samples from the hearts were homogenized in phosphate-buffered saline (100 mM, pH 7.4, 1:5 *w*/*v*) and then were centrifuged at 4 °C for 10 min 10,000× *g*; the supernatants were then used for measuring H_2_S and NOx levels. The methylene blue method was used for measuring H_2_S [78]; details can be found elsewhere [38]. This method overestimates H_2_S levels as it measures free H_2_S, HS^−^ (hydrosulfide anion), and S^2−^ (sulfide) [79,80]. Therefore, our results presented here indicate the sum total of these species. In addition, NOx concentrations in the heart tissue were measured by the Griess method [81] using a commercial kit (Pazhoheshkave Kav Afagh, Tehran, Iran). Intra-assay coefficient of variation for H_2_S and NOx in the hearts were 3.2% and 2.9%, respectively. In addition, concentrations of total protein in isolated hearts were measured using the Bradford method [82], and results for H_2_S and NOx are reported based on nmol/mg protein.

### 4.5. Measurement of Infarct Size

At the end of the reperfusion period, infarct size was measured as previously described [83]. In brief, the frozen heart samples were cut into thin slices and incubated in 2, 3, 5-triphenyltetrazolium chloride (1% in phosphate buffer solution, 20 mM, pH 7.4) at 37 °C for 10 min. The slices were immersed in 10% formalin for 24 h to identify viable myocardium (red color) from necrotic tissue (gray color). The infarct size for each heart was analyzed by Photoshop CS6 software and expressed as percentage of the total area.

### 4.6. Measurement of mRNA Expression

Details of RNA extraction, cDNA synthase, and qRT-PCR have been previously reported [84]. Total RNA was extracted from 10 mg of rat heart tissue with the RNX-Plus solution kit (Cinagen Co., Tehran, Iran). cDNA synthesis was performed using Thermo Scientific RevertAid Reverse Transcriptase in accordance with the manufacturers’ instructions. Primers were designed using primer3 and Gene Runner; primer sequences employed are shown in Table 3. Amplifications were performed in a Rotor Gene 6000 real-time PCR machine (Corbett, Life science, Sydney, Australia). Target genes were normalized with ß-actin as reference. Fold changes in mRNA expression for CSE, CBS, 3-MST, eNOS, iNOS, nNOS, RKIP, and NF-κB genes were calculated by the $2^{-\Delta \Delta C_{t}}$ method.

### 4.7. Measurement of Oxidative Stress Indices in the Heart Tissue

Measurement of MDA concentration, CAT activity, TAC concentration, as well as GSH and GSSG + GSH concentration were done by the method of Satoh [85], the method of Hadwan [86], ferric reducing/antioxidant power (FRAP) assay [87], and the method of Sedlak and Lindsay [88], respectively; details of measurement have been previously reported [38]. Intra-assay coefficients of variation for MDA, CAT, TAC, and GSH were 3.6%, 2.5%, 0.77%, and 1.7%, respectively.

### 4.8. Statistical Analyses

Data were analyzed using GraphPad Prism software (Version 6, La Jolla, San Diego CA, USA), values are expressed as mean ± SEM. To compare the body weights at the start and the end of study, body weight gain, heart weight, heart weight/body weight, systolic blood pressure, H_2_S level, NOx level, oxidative stress indices, baseline hemodynamic parameters (LVDP, heart rate, and ±dp/dt), and infarct size between groups, one-way analysis of variance (ANOVA) followed by the Bonferroni post hoc test was used. For analyzing the data for heart rate, LVDP and ±dp/dt during the IR period between groups, two-way mixed (between-within) ANOVA, followed by the Bonferroni post hoc test was used. The Mann-Whitney U test was used for comparing fold changes in mRNA expression of CSE, CBS, 3-MST, eNOS, iNOS, nNOS, RKIP, and NF-κB genes between groups. Two-sided *p*-values < 0.05 were considered statistically significant.

## 5. Conclusions

**Figure 10:**
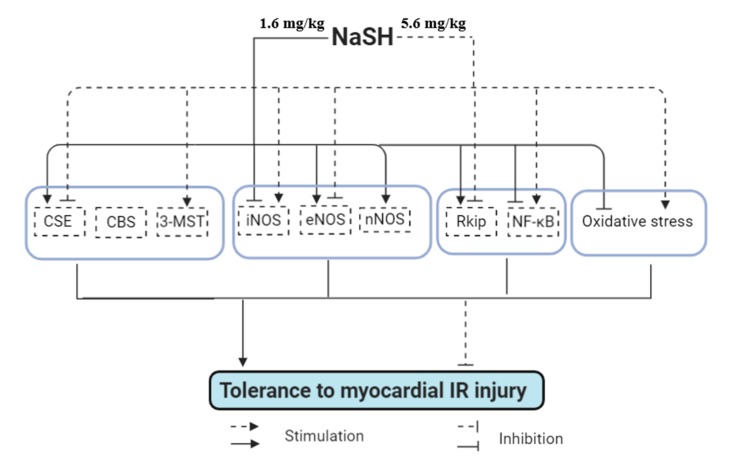
Proposed mechanism for the protective and detrimental effects of NaSH against myocardial IR injury in normal rat. Solid and dashed arrows indicate effect of NaSH administration at 1.6 mg/kg and 5.6 mg/kg, respectively. Standard arrows indicate upregulation while flat-headed arrows indicate downregulation. CSE, cystathionine γ-lyase; CBS, cystathionine β-synthase; 3-MST, 3-mercaptopyruvate sulfurtransferase; NOS, nitric oxide synthase; nNOS, neuronal NOS; eNOS, endothelial NOS; iNOS, inducible NOS; RKIP, Raf kinase inhibitor protein; NF-κB, nuclear factor kappa-B.

As illustrated in Figure 10, NaSH exhibited biphasic effects in our study, i.e., low dose had no effect, intermediate dose had a protective effect, whereas a high dose exacerbated myocardial IR injury. Higher tolerance to IR injury in hearts isolated from rats treated with intermediate dose of NaSH, at least in part, was associated with higher CSE-derived H_2_S and lower iNOS-derived NO as well as lower oxidative stress in the heart tissue after IR. In addition, the beneficial effects of H_2_S was accompanied with a decrease in NF-κB expression and increase in RKIP expression. NaSH at a high dose (5.6 mg/kg) had the opposite effects.

## Figures and Tables

**Table 1:** Effects of different doses of NaSH on body and heart weights in normal Wistar rats.

Parameters	Groups					
	Control	NaSH (mg/kg/day)				
		0.28	0.56	1.6	2.8	5.6
Initial body weight (g)	239.7 ± 2.8	238.0 ± 2.2	245.7 ± 2.5	241.7 ± 1.9	245.2 ± 1.6	242.8 ± 2.2
Final body weight (g)	291.3 ± 4.6	290.5 ± 3.3	290.2 ± 2.6	295.7 ± 2.2	299.8 ± 2.5	291.0 ± 2.7
Body weight gain (g)	51.67 ± 3.8	52.5 ± 5.2	44.5 ± 3.9	54.0 ± 3.6	54.7 ± 2.8	48.2 ± 2.3
Heart weight (g)	1.01 ± 0.05	1.01 ± 0.05	0.99 ± 0.40	1.10 ± 0.05	1.10 ± 0.04	1.37 ± 0.06 *
Heart weight/Body weight (%)	0.35 ± 0.02	0.35 ± 0.02	0.34 ± 0.01	0.37 ± 0.02	0.37 ± 0.01	0.47 ± 0.02 *

* Statistically significant difference compared to non-treated control rats. Values are mean ±SEM (n = 6/each group).

**Table 2:** Summary of studies indicating protective in vivo effect of NaSH administration on myocardial IR injury in normal rats *.

Study	Year	Rat strain	Dose (μmol/kg) #	Duration **	Mechanism	Administration Route	Ref.
Geng et al.	2004	Wistar	2.8 and 14	5 days	Inhibition of oxidative stress	Daily IP injection	[42]
Sivarajah et al.	2006	Wistar	50	15 min	Opening of mitochondrial K_ATP_ channels	Single IV injection	[31]
Zhu et al.	2007	Wistar	14	7 day	Elevation of H_2_S concentrations	Daily IP injection	[30]
Zhu et al.	2008	Sprague–Dawley	2.8 and 14	20 min	Inhibition of apoptosis	Single IV injection	[44]
Zhuo et al.	2009	Wistar	14	6 days	Inhibition of apoptosis	Daily IP injection	[32]
Sivarajah et al.	2009	Wistar	50	15 min	Inhibition of apoptosis and inflammation	Single IV injection	[24]
Pan et al.	2009	Sprague–Dawley	0.1, 1, 3, 10 and 30 †	1 day	Activation of protein kinase C	Single IP injection	[45]
Yao et al.	2010	Sprague–Dawley	1, 10, 30, 100, and 300 ‡	10 min	Inhibition of apoptosis	Single IV injection	[40]
Yao et al.	2012	Wistar	14	7 days	Inhibition of apoptosis	Daily IP injection	[29]
Issa et al.	2013	Wistar	3.57	10 min	Inhibition of inflammation and iNOS expression and activation of Akt/eNOS pathway	Single IV injection	[18]
Li et al.	2015	Sprague–Dawley	1.4, 2.8, and 14	10 min	Inhibition of endo/sarcoplasmic reticulum stress	Single IV injection	[46]
Kang et al.	2017	Sprague–Dawley	30	30 min	Inhibition of apoptosis	Single IP injection	[28]

* All studies have been conducted in male rats; ** duration of administration before ischemia; † protective effects have been observed for 0.1, 1, 3, and 10 μmol/kg but not for 30 μmol/kg; ‡ protective effect has been observed only at 30 μmol/kg; # to convert from μmol/kg to mg/kg multiply by 0.056; IP, intraperitoneal; IV, intravenous.

**Table 3:** Primers used for real-time PCR analysis.

Primer Name	Gene bank Accession No.	Primer Sequence (5′→3’)
CSE	NM_017074.1	Forward: TTGTATACAGCCGCTCTGGA Reverse: CGAGCGAAGGTCAAACAGTG
CBS	NM_012522.2	Forward: TGGTGACTCTCGGGAACATG Reverse: AGGTGGATCGGCTTGAACTG
3-MST	NM_138843.1	Forward: GGCATCGAACCTGGACACATC Reverse: ACTGGCGTTGGATCTCCTCTG
iNOS	NM_012611	Forward: ACCATGGAGCATCCCAAGTA Reverse: CAGCGCATACCACTTCAGC
eNOS	NM_021838.2	Forward: TGACCCTCACCGATACAACA Reverse: CGGGTGTCTAGATCCATGC
*nNOS*	NM_052799.1	Forward: AATCTCAGGTCGGCCATCAC Reverse: ATCCCCCAAGGTAGAGCCAT
RKIP	NM_017236.1	Forward: ACTTCCTGGTGGTCAACATGAA Reverse: TCCGGAGCCCACGTATTC
NF-κB p50	NM_001276711.1	Forward: AGAGGATGTGGGGTTTCAGG Reverse: GCTGAGCATGAAGGTGGATG
ß-actin	NM_031144.3	Forward: GCGTCCACCCGCGAGTACAAC Reverse: CGACGACGAGCGCAGCGATA

CSE, cystathionine γ-lyase; CBS, cystathionine β-synthase; 3-MST, 3-mercaptopyruvate sulfurtransferase; NOS, nitric oxide synthase; iNOS, inducible NOS; eNOS, endothelial NOS; nNOS, neuronal NOS; RKIP, Raf kinase inhibitor protein; NF-κB, nuclear factor kappa-B.
